# Factors Influencing Repurchase Intention in Drive-Through Fast Food: A Structural Equation Modeling Approach

**DOI:** 10.3390/foods10061205

**Published:** 2021-05-27

**Authors:** Yogi Tri Prasetyo, Allysa Mae Castillo, Louie John Salonga, John Allen Sia, Thanatorn Chuenyindee, Michael Nayat Young, Satria Fadil Persada, Bobby Ardiansyah Miraja, Anak Agung Ngurah Perwira Redi

**Affiliations:** 1 School of Industrial Engineering and Engineering Management, Mapúa University, 658 Muralla St., Intramuros, Manila 1002, Philippines; maecastillo01@gmail.com (A.M.C.); louiejohnsalonga@gmail.com (L.J.S.); allensia4500@gmail.com (J.A.S.); thanatorn@webmail.npnu.ac.th (T.C.); mnyoung@mapua.edu.ph (M.N.Y.); 2 School of Graduate Studies, Mapúa University, 658 Muralla St., Intramuros, Manila 1002, Philippines; 3 Logistics and Supply Chain Management Program, Nakhon Pathom Rajabhat University, Nakhon Pathom 73000, Thailand; 4 Department of Business Management, Institut Teknologi Sepuluh November, Kampus ITS Sukolilo, Surabaya 60111, Indonesia; satriafp@gmail.com (S.F.P.); bobard.m@outlook.com (B.A.M.); 5 BINUS Graduate Program—Master of Industrial Engineering, Industrial Engineering Department, Bina Nusantara University, Jakarta 11480, Indonesia; wira.redi@binus.edu

**Keywords:** drive-through, fast food, structural equation modeling, extended elaboration-intrusion theory, repurchase intention

## Abstract

The drive-through fast-food industry has been one of the fastest businesses growing over the past decades in developing countries, including the Philippines. The purpose of this study was to evaluate factors influencing costumers’ repurchase intention in a drive-through fast food in the Philippines by utilizing the structural equation modeling (SEM) approach. A total of 305 Filipinos answered the online questionnaire, which contained 38 questions. The results of SEM indicated that subjective appetite (SA) was found to have a significant direct effect on menu options (MO). Consequently, MO was found to have significant direct effects on imagery elaboration (IE), vividness (VV), and convenience (CO), and an indirect effect on order accuracy (OA). Finally, SA, MO, IE, VV, OA, and CO were found to have significant effects on satisfaction (S), which subsequently led to loyalty (L) and repurchase intention (RI). Interestingly, MO was found to have the highest indirect effect on RI, indicating that MO is an important consideration for RI. This is the first comprehensive study evaluating drive-through fast food in the Philippines. The causal relationships of the present study can be applied and extended to evaluate the repurchase intention of drive-through fast food in other countries.

## 1. Introduction

Drive through service has been an important part of foodservice business research. It is a common feature of fast-food restaurants (FFRs). The primary goal of these drive-through services is to provide fast and convenient service to the customer [1] while increasing the number of customers that can be served, as opposed to the traditional walk-in transactions. Usually, drive-through systems are located on the assigned drive-through lane, remotely from the building. Upon entering the drive-through lane, the customer stops his vehicle momentarily to choose an order from the food offered by the restaurant on the menu.

Thus, a menu acts as the first point of interaction for customers in the restaurants, providing cues about their impending encounter, while, simultaneously, establishing the restaurant’s strategic marketing plan, personality, and brand identity [2,3]. A well-designated menu can facilitate restaurants’ sales by steering the attention of customers to a specific menu item offered. Thomas and Mills [4] mentioned that few studies empirically assessed how menu information gathered by restaurants influences the attitudes of the customers towards the restaurant, including their consequent behavioral intentions.

In the Philippines, the fast-food industry (FFI) has been one of the fastest-growing business sectors over the past decade. According to a report by the Philippine Statistics Authority (Table 1), FFI generated the highest income of PHP 203 billion, with 4411 establishments in 2016. Local brands have transformed FFRs as pioneers and expanded their franchises into other countries all around the world. [5]. Aside from Korea, the Philippines is the only country where McDonald’s is not the leading and most successful company in the fast-food industry (FFI), as Filipino fast-food market (FFM) prioritized localization while retaining the prestige of foreign brands [5,6].

Jollibee is a local brand that operates similar to a Filipino version of McDonald’s [6], providing both rice dishes, a daily meal for locals citizens, and fast food products typically sold in the United States. As of November 2019, Jollibee currently holds a dominating 58% share of the FFM in the Philippines, with a total of 1150 outlets nationwide, outnumbering its competitor, McDonald’s, with more than 640 stores. Interestingly, part of Jollibee’s success stems from imitating the innovative principles and techniques pioneered by McDonald’s in the United States decades ago.

Despite the customers’ familiarity with order selection, there is a significant lack of academic research in the Philippines that addresses the relative importance of attributes affecting repurchase intention in drive-through fast food. Bowen and Morris [7] mentioned that the representational quality of the products listed on a menu, as a unique type of marketing situation is, comparable to a professional speech delivery. In addition, McCall and Lynn [3] highlight the relative importance of the role of restaurant menus suggesting that the menu drives a process that might entice a diner to enter a restaurant. According to Namkung and Jang [7], increased competition in the global FFRs has made customer satisfaction more important in order to build customer loyalty and to enhance business performance. Further studies are required to verify the extent of what influences customer satisfaction and repurchase intentions in a drive-through setting.

This research aims to evaluate factors influencing costumers’ repurchase intention in a drive-through fast food in the Philippines by utilizing the structural equation modeling (SEM) approach. Following some previous studies [8,9,10], SEM is used to analyze the causal relationships between measured variables and latent constructs. The causal relationships in this study can be applied and extended to evaluate the repurchase intention of drive-through fast food in other countries.

## 2. Theoretical Research Framework

**Figure 1:**
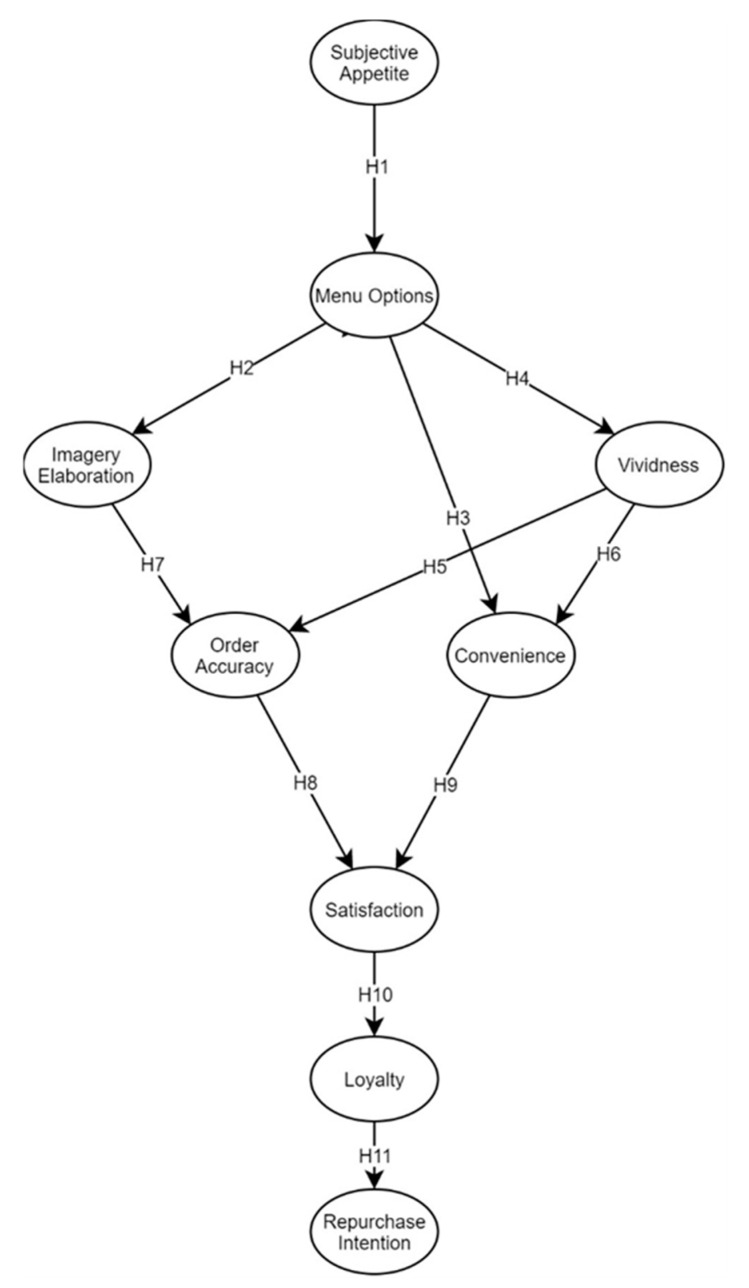
Theoretical research framework.

Figure 1 represents the theoretical research framework of the current study. The theoretical basis of the current model was derived mainly from Peters and Remaud’s [11] and other several studies. The model is developed from the factors of vividness, subjective appetite, and imagery elaboration, as investigated by Lee and Kim [12], and from the customer perceptions analysis of service performance, carried out by Cronin and Taylor [13]. The current model also incorporates factors such as value for money [14], convenience [15], taste preference [16], introducing latent variables such as menu options and order accuracy as well.

Meanwhile, the entire area of food intake, choice, motivation, and preference is covered by subjective appetite [17]. The sight of preferred or less preferred is sufficient enough to influence the customer’s desire to eat [18]. Consequently, a study by Schulte-Mecklenbeck et al. [19] stated that customers who are aware of their food options commonly ignore complex information on the menu when ordering. Thus, we hypothesized that:

**Hypothesis** **1** **(H1).** *Subjective appetite of customers had a significant direct effect on the menu options offered by the drive-through.*

Menu options can be presented in many ways and are crucial for restaurants’ branding because it is an internal advertisement component for informing customers about their impending dining experience [20]. Mitchell et al. [21], suggested that mental imagery and the manifestation of mental imagery have a positive correlation through cognitive style. In addition, the past study also stated that mental imagery exhibits a significant correlation with menu presentation [22]. Hence, customers can easily associate menu items with appetite if menus elicit a sensory response. Consequently, we hypothesize that:

**Hypothesis** **2** **(H2).** *Menu options had a significant direct effect on the imagery elaboration of the menu in drive-through.*

Menu designers claim that the elements of design (e.g., copy, color, paper, typeface layout, and so on) can draw the customers’ attention to the items that restaurants want to sell [7]. Laying out the assigned item with a box, putting it in striking print, employing a larger sort estimate, and including a color photo are a few regularly prescribed ways to draw attention to the menu items, increasing sales, as a result [23]. If utilized wisely, specialists claim that these procedures can viably offer targeted items. Thus, we hypothesized that:

**Hypothesis** **3** **(H3).** *Menu options had a significant direct effect on the convenience of menus in drive-through.*

The degree to which pictures have been reflected in one’s intellect influences the conciseness of the stimuli [24]. Mental imagery has characterized as a mechanism by which tactile data are interpreted in one’s intellect, commonly conveyed by vividness, imagery amount, and imagery elaboration [22]. Vividness is the accuracy with which the person encounters a picture [25], whereas imagery elaboration is the actuation of info within the generation of imaginative illustrations above what the stimulus provides [26]. The customer can interface prior encounters to the comparing information through picture handling and tactile experiences (such as scent, taste, sight, and other sensations) in working memory [24]. Thus, we hypothesized that:

**Hypothesis** **4** **(H4).** *Menu options had an influential direct impact on the vividness of menus in the drive-through.*

Lee and Kim [12] initially attempted to study the effect of pictures and video menu presentation on the imagery perspective of the customers. Their study encompasses the relationship between vividness, imagery elaboration, imagery quantity, and subjective appetite. Hence, we proposed that:

**Hypothesis** **5** **(H5).** *Vividness had a significant direct effect on the order accuracy in drive-through.*

Convenience is defined as the decreased time and effort customers must devote to purchasing or utilizing items and services, with the help of innovation [27]. Coyle and Thorson [28] showed a high degree of vividness, contributing to a positive disposition towards the website. Correlating this information to our current study, we hypothesized that:

**Hypothesis** **6** **(H6).** *Vividness had a significant direct effect on the convenience of menu in drive-through.*

Hodes [29] stated that, in the context of learning instructions, imagery instructions appear to be effective in absorbing the information. Being related to order selection, Beldona et al. [30] suggested that an elegant menu design can cause customers to draw their attention to a certain item in the menu. Imagery elaboration enables the conveyance of information in the development of visual representations beyond what the stimulus offers [25]. The relationship between imagery elaboration and subjective appetite has been studied based upon the elaboration-intrusion (EI) theory of desire [31]. The study of Schumacher et al. [32], which stated that craving triggers of the elaboration-intrusion theory (e.g., picturing myself having the food) were significantly positive in showing craving intensity. FFRs present an image of their product to capture their customers’ attention [31]. The sensory stimuli (e.g., taste, look, and smell) are the primary thoughts of the customers while ordering the food that they want, which also become the basis for confirming accuracy after receiving the food [32]. Hence, we hypothesized the following: Hodes [29] stated that, in the context of learning instructions, imagery instructions appear to be effective in absorbing the information. Being related to order selection, Beldona et al. [30] suggested that an elegant menu design can cause customers to draw their attention to a certain item in the menu. Imagery elaboration enables information in the development of visual representations beyond what the stimulus offers [25]. The relationship between imagery elaboration and subjective appetite has been based on the elaborated intrusion (EI) theory of desire [31]. The study of Schumacher et al. [32], which stated that craving triggers the elaboration-intrusion theory (e.g., picturing myself having the food) was significantly positive in showing craving intensity. FFRs present an image of their product to capture their customers’ attention [31]. The sensory stimuli (e.g., taste, look, and smell) are the primary thoughts of the customers while ordering the food that they want, which also become the basis for confirming accuracy after receiving the food [32]. Hence, we hypothesized the following:

**Hypothesis** **7** **(H7).** *Imagery elaboration had a significant direct effect on order accuracy.*

Handling customer satisfaction in terms of order fulfillment is dependent on how the organization meets customer requirements. [33]. Order accuracy involves all operations starting with the customer’s purchase decision until the customer receives its order and the quality of the products are fully satisfied [34]. Thus, we hypothesized the following:

**Hypothesis** **8** **(H8).** *Order accuracy had a significant direct effect on satisfaction.*

According to Yale and Venkatesh [35], convenience has been broken down into time efficiency, ease of access, applicability, and avoidance of dissatisfaction. The positive views of customers toward the services and the relevance of a food company provide an increase in their satisfaction [36]. The study of Cheng et al. [37] has mentioned that convenience enhances customer satisfaction. Therefore, we hypothesized that:

**Hypothesis** **9** **(H9).** *Convenience had a significant direct effect on satisfaction.*

Namin [10] suggests that having a high level of service quality will gain satisfied customers, leading to having more loyal customers. According to Oliver [38], loyalty is described as the willingness of customers to continuously re-buy and re-patronize a chosen product or service. The previous study by Belaid and Behi [39] also mentioned that a powerful brand-loyalty predictor is satisfaction. Therefore, we proposed that:

**Hypothesis** **10** **(H10).** *Satisfaction had a significant direct effect on loyalty.*

The previous study by Yi and La [40] considered the characteristics of loyal customers by evaluating loyalty as a high proportion of the same choice of brand, a high motive of positive word-of-mouth, and a high intention to repurchase. Behavioral loyalty is defined purely as paying back the goods or services [41]. The study by Curtis et al. [41] has indicated that loyalty and repurchase intention have a strong positive relationship. Thus, we proposed that:

**Hypothesis** **11** **(H11).** *Loyalty had a significant direct effect on repurchase intention.*

## 3. Methodology

### 3.1. Participants

Following Akram et al. [9], the research instrument of this study is in the form of an online questionnaire. The chosen population consisted of those people who have had a recent experience of service in FFRs drive-through in the Philippines. A total of 305 participants willingly answered the survey questionnaire, which contains 38 questions.

Table 2 represents the descriptive statistics that were calculated to ascertain the characteristics of the sample. Among the 305 respondents, 50.16% were female and 49.84% were male. Most respondents were between 18 and 36 years of age (80.33%). About 12.79% of the survey participants were between the age of 36 and 55, 5.25% were aged below 18 years, and 1.64% were over 56 years of age. Approximately 40.66% of respondents indicated that they visit drive-through daily, 31.80% visit a few times per week, 10.16% visit a few times per month, 8.20% visit about once per week, 7.87% visit about once per month, and only 1.31% visit rarely. Most of the respondents usually visit drive-through for lunch (38.03%). About 28.85% of the respondents visit for a snack, 18.36% visit for breakfast, and only 14.75% of the respondents visit for dinner. Approximately 59.67% of the respondents indicated that they spend between PHP 200 and PHP 400 in a drive-through, 20% spend PHP 200 and less, 17.70% spend between PHP 400 and PHP 800, and only 2.62% spend over PHP 800.

### 3.2. Questionnaire

A self-administered questionnaire was developed to measure the perceptions of the customers about their recent drive-through experience in fast food. A convenience sampling method was utilized to distribute the questionnaire. The questionnaire consists of 11 sections and is supported by some previous studies: (1) demographic information (gender, age, time of visit, and so on); (2) value for money; (3) subjective appetite; (4) menu options; (5) vividness; (6) imagery elaboration; (7) convenience; (8) order accuracy; (9) satisfaction; (10) loyalty; and (11) repurchase intention (Table 3). All latent constructs were measured using 5-point Likert.

### 3.3. Structural Equation Modeling

Structural equation modeling (SEM) is a progressive multivariate procedure that focuses on the interrelationships among constructs [49]. We utilized AMOS 22 with a maximum likelihood estimation approach to derive the model. Figure 1 demonstrates that our SEM construct had nine latent variable constructs, including one exogenous latent variable (SA) and seven endogenous latent variables (MO, IE, VV, OA, CO, S, L, RI).

The model fit of the SEM was measured by six measures: goodness of fit index (GFI), adjusted goodness of fit index (AGFI), root mean square error of approximation (RMSEA), incremental fit index (IFI), Tucker–Lewis index (TLI), and comparative fit index (CFI). A value greater than 0.80 for GFI and AGFI is the minimum indication of good model fit [50]. In addition, a value lower than 0.07 is a good indication of an acceptable model for RMSEA [49]. Finally, a value greater than 0.90 for IFI, TLI, and CFI is an indication of a good model fit [49].

## 4. Results

**Figure 2:**
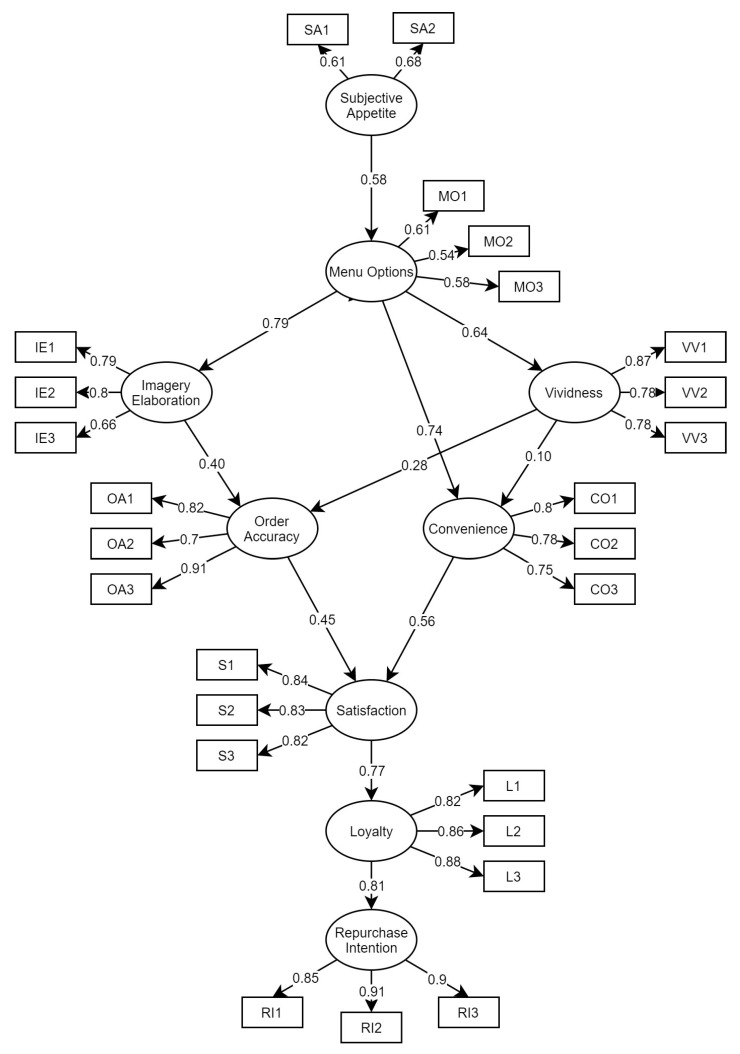
The final SEM for evaluating factors influencing customers’ repurchase intention in drive-through fast foods, in the Philippines.

Figure 2 represents the SEM for evaluating factors influencing customers’ repurchase intention in drive-through fast food, in the Philippines. In addition, Table 4 represents the construct validity and reliability of the model, which were calculated based on Cronbach’s α, average variance extracted (AVE), and composite reliability (CR). Based on this figure, all hypotheses were significant, indicating that our model construct was acceptable. Hair [49] mentioned that all of the factor loadings should be statistically significant. An ideal standardized factor loading should be higher than 0.5, and ideally 0.7 or higher [49]. If the factor loadings are lower than 0.7, they can still be considered significant; however, more variances are found in the measure than that of the explained variance [49], such as SA1 (λ:0.61), SA2 (λ:0.68), MO1 (λ:0.61), MO2 (λ:0.54), MO3 (λ:0.58), and IE3 (λ:0.66).

As presented in Table 5, the GFI and AGFI values were 0.879 and 0.851, respectively, indicating that the model passed the minimum cutoff of the model fit [50]. In addition, the RMSEA value was 0.055, indicating that it was lower than the suggested cut-off of 0.07. IFI, TLI, and CFI values were greater than the suggested cutoff of 0.90, indicating that the hypothesized construct of the specified model reflected the observed data very well. Finally, the direct effect, the indirect effect, and the total effect are presented in Table 6 as the basis for the discussion particularly related to the causal relationships among latent variables.

## 5. Discussion

In the Philippines, the fast-food industry (FFI) has been one of the fastest-growing business sectors, over the past decade. The present study applied the structural equation modeling (SEM) approach to examine the causal relationships between subjective appetite (SA), menu options (MO), vividness (VV), imagery elaboration (IE), convenience (CO), order accuracy (OA), satisfaction (S), loyalty (L), and repurchase intention (RI) of drive through fast food in the Philippines. An online survey was conducted during this study, and a total of 305 respondents were participated.

SEM indicated that SA (β: 0.58, *p* = 0.001) had a significant direct effect on MO, which makes several implications in the customers’ choices when ordering in a drive-through. While visiting a drive-through restaurant, hunger and a strong desire to eat, positively affect customers’ choice of food from the menu offered by the restaurant. Hence, management should take note that a well-designed menu should cater to all customers’ food preferences.

Regarding the impact of menu options, the current model indicated that MO had a significant effect on VV (β: 0.64, *p* = 0.001), CO (β: 0.74, *p* = 0.001), and IE (β: 0.79, *p* = 0.001), which can be interpreted in several ways. First, customers can easily find a suitable food option when the menu offered by the restaurant is clear and easy to read. Second, customers find it easier to make their food choices when the menu is located within their eye level. Third, customers’ food choices can be based on the menu’s representational quality, as it can attract customers to order food in drive-through by eliciting a sensory response. These findings were supported by the study of Bowen and Morris [7], who mentioned that the customers’ attention towards food items can be attracted by the menu presentation. Thus, management should develop a menu that can capture the customers’ attention, leading them to visit and order in their drive-throughs. Despite the results from most recent studies [10,13], MO was found to have the highest indirect effect on RI (β: 0.42, *p* = 0.001), indicating that MO is one of the most important considerations for RI. Based on the results, customers who find drive-throughs that offer more options suited for them are most likely to continue visiting them in the future.

Subsequently, the results showed that VV has a significant effect on OA (β: 0.28, *p* = 0.001) but an insignificant effect on CO (β: 0.10, *p* = 0.46). An interpretation of this result is that customers would use the menu presentation as a basis for checking the accuracy of their orders by checking the correctness and completeness of their food choices. Managers who wish to enhance their order accuracy should ensure that their staff is accurately delivering the customers’ orders based on the quality of their menu’s presentation.

Regarding satisfaction, SEM indicated that S was significantly affected by OA (β:0.45; *p* = 0.001) and CO (β:0.56; *p* = 0.001). The result implied that customers are most satisfied when the image shown on the menu board is clear and when customers receive their orders completely. Moreover, S was found to have a significant direct effect on L (β:0.77; *p* = 0.001). According to Carpenter and Fairhurst [51], the success of the company’s technique depends on its capacity to fulfill its promises to customers, which leads to building a long-term beneficial relationship. Based on the result, management should provide food with excellent quality in order to make customers satisfied with their visit to the drive-through, saying positive things about the restaurant, as a result. Thus, when customers are satisfied with their recent experience in drive-through services, they intend to encourage other people to visit the drive-through as well.

Lastly, the SEM proved that there was a positive interrelation between L and RI (β:0.81; *p* = 0.001), which favors the previous studies’ theoretical research framework. The desire to purchase again [52] and the desire to indulge in good referral are two ways of ensuring consumers’ intention to buy again. Excellent service would develop better word-of-mouth and continuously improve the customers’ experience. Based on the results of the current study, customers who are willing to say positive things and recommend drive-through to other people are most likely to continue visiting the drive-through in the future, regardless of how often they visit.

### 5.1. Managerial Implications

The results of the research would help Philippine fast-food restaurants to assess the factors influencing customers’ repurchase from their drive-throughs. First, the researchers discussed the significant effect of SA on MO in a drive-through fast food. The results showed the willingness of the customers to buy food from the fast-food since it is inexpensive, and the food quality is acceptable. Hence, different drive-through fast food restaurants must ensure that the menu is attractive to the customers. Providing a well-designed menu will entice customers and excite them to order. Secondly, the importance of MO can be recognized by the positive effect on VV, CO, and IE for drive-through fast-food customers. The finding provides the insightful implication of imagery and vividness to improve the design of the menu. Customers much prefer ordering in a drive-through fast food because it is more convenient and inexpensive, and it tastes good. Companies who are not yet familiar with enhancing their menu should invest time in upgrading their menu. Developing menu designs is one of the most critical strategic campaigns of a restaurant to keeps the brand fresh in the customer’s mind and to increase profitability while also maintaining a competitive edge.

### 5.2. Limitations and Future Research Direction

Although this research provides important evidence of the factors influencing customers’ repurchase intention in drive-through fast food in the Philippines, the authors would like to acknowledge several limitations on the SEM of the current study. First, the data from the current study were collected from Filipino customers who had a recent experience in drive-through. Thus, it may not be generalized for all nationalities because of their different cultures. In other words, customer motives can differ based on their taste preference, and other countries offer different menus to cater to their local palates. Future work should cross-validate the existing paradigm for new ideas into how cultural influences regulate hierarchical interactions in different ways and across countries. Second, the results from the derived SEM could have different results for electronic, digital menus with varying interactive features, whereas the current study only focused on traditional menus [12]. Future studies should also incorporate food quality and customers’ complaints as one of the key constructs in the current SEM model to reveal more information regarding their interrelationship with loyalty and satisfaction. Furthermore, when evaluating the role of intentions as a connection between satisfaction and repurchase of actions, future studies are recommended to consider two specific intention constructs, such as intentions as expectations and intentions as preferences to the current SEM. Finally, there are several additional factors that could probably enhance satisfaction such as Wi-Fi services [53], cleanliness, and even payment options (cash, debit card, and e-money). Future research can add these additional factors in the model.

## 6. Conclusions

Over the past decades, drive-through fast-food industry has been one of the fastest growing businesses in the Philippines. According to the Philippines Statistics Authority, the fast-food industry has generated the highest income of PHP 203 billion with 4411 establishments in 2016. The purpose of this study was to evaluate factors influencing costumers repurchase intention in a drive-through fast food in the Philippines by utilizing the structural equation modeling approach [54,55,56,57,58,59]. A total of 305 Filipinos answered the online questionnaire, which contained 38 questions. The results of SEM indicated that subjective appetite (SA) was found to have significant direct effects on menu options (MO). Consequently, MO was found to have significant direct effects on imagery elaboration (IE), vividness (VV), and convenience (CO), and an indirect effect on order accuracy (OA). Finally, SA, MO, IE, VV, OA, and CO were found to have significant effects on satisfaction (S), which subsequently lead to loyalty (L) and repurchase intention (RI). Interestingly, MO was found to have the highest indirect effect on RI, indicating that MO is one important consideration for RI. The results of the present study can be applied and extended to evaluate the repurchase intention of drive-through fast food in other countries.

## Figures and Tables

**Table 1:** Accommodation and food service activities establishments by industry in the Philippines, 2016.

2009 PSIC Code	Industry Description	Number of Establishments	Employment as of November 15th		Total Income
			Total	Paid Employees	
		(1)	(2)	(3)	(4)
	Accommodation and Food Service Activities	30,889	495,973	485,422	551,083,110
I55101	Hotels and motels	2767	69,828	69,026	90,171,024
I55102	Resort hotels	1112	32,683	31,884	29,583,591
I55103	Condotels	56	995	995	1,348,929
I55104	Pension houses	399	3104	2881	1,202,641
I55105	Camping sites/facilities	7	198	198	146,421
I55109	Other short term accommodation activities, n.e.c.	124	985	856	706,546
I55901	Dormitories/boarding houses	499	3030	3030	1,871,232
I55909	Other accommodation, n.e.c.	29	144	142	72,063
I56101	Restaurants	7218	130,965	129,521	129,761,811
I56102	Fast-food chains	4411	138,051	137,002	203,007,168
I56103	Cafeterias	4725	33,854	30,204	27,859,684
I56104	Refreshment stands, kiosks, and counters	4209	33,332	32,986	25,857,313
I56109	Other restaurants and mobile food service activities, n.e.c.	1679	5566	5275	9,513,724
I56210	Event catering	442	7517	7324	7,395,711
I56290	Other food service activities	340	2396	2396	1,131,806
I56301	Night clubs	115	1726	1609	969,143
I56302	Bars and cocktail lounges	1548	19,405	18,481	11,252,945
I56303	Café or coffee shops	947	10,991	10,509	7,912,984
I56302	Other beverage serving activities, n.e.c.	262	1104	1104	1,318,373

Source: https://psa.gov.ph/content/2016-annual-survey-philippine-business-and-industry-aspbi-accommodation-and-food-service-0 (accessed on 20 May 2021).

**Table 2:** Descriptive statistics of the respondents (*N* = 305).

Characteristics	Category	*N*	%
Gender	Male	152	49.84
	Female	153	50.16
Age	Below 18	16	5.25
	18–36	245	80.33
	37–55	39	12.79
	Over 56	5	1.64
Frequency of Visit	Daily	124	40.66
	A few times per week	97	31.8
	About once per week	25	8.2
	A few times per month	31	10.16
	About once a month	24	7.87
	Rarely	4	1.31
Time of Visit	Breakfast	56	18.36
	Lunch	116	38.03
	Snack	88	28.85
	Dinner	45	14.75
Money Spent in Drive-through	PHP 200 and Below	61	20
	PHP 200–PHP 400	182	59.67
	PHP 400–PHP 800	54	17.7
	PHP 800 and above	8	2.62

**Table 3:** The constructs and measurement items included in the questionnaire.

Latent Variables	Items	Measures	Supporting References
Subjective Appetite (SA)	SA1	I was hungry during my last drive-through visit.	Lee and Kim [12]
	SA2	I have a strong desire to eat during my last drive-through visit.	
Menu Options (MO)	MO1	The fast food’s drive through has more options suited for me.	
	MO2	My preferred food was available during the time of my visit.	
	MO3	I could find a suitable option easily.	
Imagery Elaboration (IE)	IE1	I imagined what the food would taste like.	Lee and Kim [12]
	IE2	I imagined the smell of the food.	
	IE3	I imagined what the actual food would look like.	
Vividness (VV)	V1	The imagery shown in the menu board was clear.	Lee and Kim [12]
	V2	The imagery shown in the menu board was detailed.	
	V3	The imagery shown in the menu board was vivid.	
Convenience (CO)	CO1	The menu board was easy to read.	Rydell et al. [15]
	CO2	The food items were easy to locate.	
	CO3	The menu board is located within my eye level.	
Order Accuracy (OA)	OA1	I received the meal I ordered correctly.	
	OA2	The staff repeats my order for recap.	-
	OA3	I received the meal I ordered completely.	
Satisfaction (S)	S1	The quality of food was excellent.	Mcneil and Young [42]; Ryu et al. [43];
	S2	The service I receive has worked out as well as I thought it would.	
	S3	I am satisfied with my decision to visit the drive-through.	
Loyalty (L)	L1	I will recommend this drive-through to other people who seek my advice.	Gallarza-Granizo et al., [44]; Wu and Mohi [45]
	L2	I will say positive things to my friends about this drive-through.	
	L3	I will encourage other people to visit this drive-through.	
Repurchase Intention (RI)	RI1	I will keep visiting the drive-through in the future.	Dipietro et al. [46]; Konuk, F.A. [47]; Liu et al. [48]
	RI2	I am looking forward to visit drive-through.	
	RI3	Regardless of how often I visit drive-through, I always look forward to visiting it again.	

**Table 4:** The construct’s validity and reliability.

Latent Variables	Items	Cronbach’s α	Factor Loadings	Average Variance Extracted (AVE)	Composite Reliability (CR)
Subjective Appetite (SA)	SA1	0.581	0.61	0.417	0.588
	SA2		0.68		
Menu Options (MO)	MO1	0.769	0.61	0.333	0.599
	MO2		0.54		
	MO3		0.58		
Imagery Elaboration (IE)	IE1	0.796	0.79	0.567	0.796
	IE2		0.80		
	IE3		0.66		
Vividness (VV)	V1	0.824	0.87	0.658	0.852
	V2		0.78		
	V3		0.78		
Convenience (CO)	CO1	0.823	0.80	0.604	0.820
	CO2		0.78		
	CO3		0.75		
Order Accuracy (OA)	OA1	0.850	0.82	0.664	0.854
	OA2		0.70		
	OA3		0.91		
Satisfaction (S)	S1	0.891	0.84	0.689	0.869
	S2		0.83		
	S3		0.82		
Loyalty (L)	L1	0.891	0.82	0.729	0.890
	L2		0.86		
	L3		0.88		
Repurchase Intention (RI)	RI1	0.917	0.85	0.787	0.917
	RI2		0.91		
	RI3		0.90		

**Table 5:** The model fit.

Goodness of Fit Measures of the SEM	Parameter Estimates	Minimum Cut-Off	Recommended by
Goodness of Fit Index (GFI)	0.879	>0.80	[50]
Adjusted Goodness of Fit Index (AGFI)	0.851	>0.80	[50]
Root Mean Square Error of Approximation (RMSEA)	0.055	<0.07	[49]
Incremental Fit Index (IFI)	0.946	>0.90	[49]
Tucker Lewis Index (TLI)	0.938	>0.90	[49]
Comparative Fit Index (CFI)	0.945	>0.90	[49]

**Table 6:** Direct effect, indirect effect, and total effects. “-” means “no path”.

Variables	Direct Effect	*p* Value	Indirect Effect	*p* Value	Total Effect	*p* Value
SA → MO	0.58	0.001	-	-	0.58	0.001
SA → VV	-	-	0.38	0.001	0.38	0.001
SA → IE	-	-	0.46	0.001	0.46	0.001
SA → CO	-	-	0.47	0.000	0.47	0.000
SA → OA	-	-	0.29	0.001	0.29	0.001
SA → S	-	-	0.39	0.001	0.39	0.001
SA → L	-	-	0.30	0.001	0.30	0.001
SA → RI	-	-	0.24	0.001	0.24	0.001
MO → V	0.64	0.001	-	-	0.64	0.001
MO → IE	0.79	0.001	-	-	0.79	0.001
MO → CO	0.74	0.001	0.07	0.44	0.81	0.001
MO → OA	-	-	0.49	0.001	0.49	0.001
MO → S	-	-	0.68	0.001	0.68	0.001
MO → L	-	-	0.52	0.001	0.52	0.001
MO → RI	-	-	0.42	0.001	0.42	0.001
VV → CO	0.10	0.46	-	-	0.10	0.46
VV → OA	0.28	0.002	-	-	0.28	0.002
VV → S	-	-	0.18	0.05	0.18	0.05
VV → L	-	-	0.14	0.05	0.14	0.05
VV → RI	-	-	0.11	0.04	0.11	0.04
IE → OA	0.40	0.001	-	-	0.40	0.001
IE → SA	-	-	0.18	0.001	0.18	0.001
IE → L	-	-	0.14	0.001	0.14	0.001
IE → RI	-	-	0.11	0.001	0.11	0.001
CO → SA	0.56	0.001	-	-	0.56	0.001
CO → L	-	-	0.43	0.001	0.43	0.001
CO → RI	-	-	0.35	0.000	0.35	0.000
OA → SA	0.45	0.001	-	-	0.45	0.001
OA → L	-	-	0.34	0.001	0.34	0.001
OA → RI	-	-	0.28	0.001	0.28	0.001
S → L	0.77	0.001	-	-	0.77	0.001
S → RI	-	-	0.62	0.001	0.62	0.001
L → RI	0.81	0.001	-	-	0.81	0.001

## Data Availability

The data presented in this study are available upon request from the corresponding author.
